# Musculoskeletal disorders in shipyard industry: prevalence, health care use, and absenteeism

**DOI:** 10.1186/1471-2474-7-88

**Published:** 2006-11-24

**Authors:** Evangelos C Alexopoulos, Dimitra Tanagra, Eleni Konstantinou, Alex Burdorf

**Affiliations:** 1 Occupational Health Department, Hellenic Shipyards SA, Athens, Greece; 2 Public Health Department, Erasmus MC, Rotterdam, The Netherlands

## Abstract

**Background:**

It is unclear whether the well-known risk factors for the occurrence of musculoskeletal disorders (MSD) also play an important role in the determining consequences of MSD in terms of sickness absence and health care use.

**Methods:**

A cross-sectional study was conducted among 853 shipyard employees. Data were collected by questionnaire on physical and psychosocial workload, need for recovery, perceived general health, occurrence of musculoskeletal complaints, and health care use during the past year. Retrospective data on absenteeism were also available from the company register.

**Results:**

In total, 37%, 22%, and 15% of employees reported complaints of low back, shoulder/neck, and hand/wrist during the past 12 months, respectively. Among all employees with at least one MSD, 27% visited a physician at least once and 20% took at least one period of sick leave. Various individual and work-related factors were associated with the occurrence of MSD. Health care use and absenteeism were strongest influenced by chronicity of musculoskeletal complaints and comorbidity with other musculoskeletal complaints and, to a lesser extent, by work-related factors.

**Conclusion:**

In programmes aimed at preventing the unfavourable consequences of MSD in terms of sickness absence and health care use it is important to identify the (individual) factors that determine the development of chronicity of complaints. These factors may differ from the well-know risk factors for the occurrence of MSD that are targeted in primary prevention.

## Background

Musculoskeletal diseases are a major cause of diminished work capabilities of industrial workers with substantial financial consequences due to workers' compensation, medical expenses, and productivity losses [1,2]. It has been estimated that the indirect costs of one workday lost due to sickness absence amount to €450 ($525; £300) [3].

Various epidemiologic studies have demonstrated that specific work-related risk factors may cause musculoskeletal complaints, but studies on primary prevention programmes often have difficulties in demonstrating that a reduction in these risk factors has resulted in a reduced occurrence of musculoskeletal complaints [4]. In addition, there is some evidence that subjects most likely to develop back pain are less likely to participate in primary preventive programs [5-7]. In the recent European guidelines for low back pain (LBP) it is argued that LBP management should focus less on the prevention of the onset of LBP episodes and more on limiting the consequences of LBP in terms of functional limitations, quality of life, and sickness absence [8]. The rationale presented is that given the high prevalence of LBP it is almost inevitable that more persons are affected by LBP at some time in their life, but most patients will recover within a few weeks. Hence, it seems more important to prevent the aggravation of LBP into chronic and disabling LBP and, thus, address the consequences such as sickness absence and health care use [9,10]. Several studies have shown that work-related risk factors as well as severity of complaints may prompt the decision among workers with musculoskeletal complaints to take sickleave [10-12]. Chronicity, pain severity, psychosocial factors, poor perceived health and musculoskeletal comorbidity have been associated with the decision to seek care [13-16]. More insight into the reasons to seek care is important because the health care received early after onset of complaints is an important predictor of long-term outcomes [17].

In the shipyard industry, workspace environments often include the well-established risk factors for predisposing low back pain and other musculoskeletal disorders (MSDs). Many workers (e.g. carpenters, plumbers, welders, mechanics, and others) are often required to adopt awkward postures such as kneeling, stooping, squatting, or lying down, for significant periods of the workday. A high prevalence of work-related MSDs has been reported among workers involved with manual materials handling, unusual and restricted postures, repetitive and static work, vibration, and poor psychological and social conditions [9,18-21]. In the Greek industry no study so far has investigated the determinants of MSDs and their consequences for sickness absence and health care use. In a previous study we confirmed the occurrence of musculoskeletal disorders in shipyard industry, with employees reporting low back pain as the most prevalent health complaint [22].

The aim of this cross-sectional study was to describe the prevalence of complaints of low back, shoulder/neck, and hand/wrist and the consequences for sickness absence and health care use and to investigate the importance of individual and work related physical and psychosocial factors for occurrence of MSDs and subsequent sickness absence and health care use. Although it must be acknowledged that this cross-sectional study has strict limitations with regard to causality, the results are nevertheless of importance to prioritize further research in this industry to improve occupational health care.

## Methods

### Study population

Baseline data were collected through questionnaires in the period between November 2003 and March 2004. Throughout this period, employees were asked during the routine bi-annual check up by the occupational health department to participate in the study by giving their informed consent. The response was 98.5% (919/933 employees). Given the study design with questions on events in the past 12 months, workers were only eligible for the current study when they had at least 1 year of work experience in the current position. Hence, the final inclusion in the study comprised 853 subjects (93% of responders).

The study population consisted of 624 (73.2%) blue collar and 229 (26.8%) white collar workers. Blue collars mainly consisted of metal workers (47%) (e.g. platters, fitters, pipe fitters), welders (15%), drivers/crane operators (10%), carpenters (8%), electricians (7%), sandblasters/painters (6%), and a variety of other jobs. White collars consisted mainly of office employees like accountants, designers, secretaries, telephone operators, computer experts, managers, and construction engineers.

### Study design and data collection

This cross-sectional study used a self-administered questionnaire that involved information on the respondent's job history, individual characteristics, physical and psychosocial risk factors at work, general health status, occurrence of musculoskeletal complaints, and health care use. Musculoskeletal complaints were ascertained by the standardized Nordic questionnaire, which has recently been translated into Greek and evaluated for its validity [23,24]. The questionnaire was tested for comprehensibility and relevance among nurses and dentists in previous studies [25,26].

Individual characteristics and work history included questions on age, anthropometry, gender, family situation, level of education, duration of employment, and previous jobs held. Personal psychological factors were not included in this study. Questions on physical work load concerned repetitive movements, awkward working postures with a bend or twisted back, prolonged sitting or standing, and strenuous arm positions like applying force with arms or hands or working with elevated arms, and use of vibrating tools. A four-point scale was used with ratings 'seldom or never', 'now and then', 'often', and 'always' during a regular workday. The answers 'often' and 'always' were classified as high exposure [25,26]. The study subjects also rated their perceived exertion on a Borg-scale ranging from 6 (very light) till 20 (very heavy), with a score of 16 or higher regarded as high perceived exertion [27].

Psychosocial aspects at work distinguished two principal areas: demands, and control [28]. Job demands were measured by 10 questions related to items such as working fast and hard, excessive work, insufficient time to complete a duty, or conflicting demands. Lack of control (decision latitude) was measured by 10 questions with six items on skill discretion and 4 items on decision authority, addressing topics such as creativity, skills, task variety, learning new things, and amount of repetitive work. We did not use the support component in the Demand-Control-Support model (co-worker and supervisor support) because in previous studies these items raised suspicious feelings among workers and affected participation. All questions were scored on a four point scale and within each domain a sum score was calculated. The demand and control sum scores were expressed as percentage of the highest possible score, with 0% indicating the best possible situation and 100% the worst possible situation. In the statistical analysis, scores above the median value were considered as the presence of a psychosocial risk.

The health status of each subject was ascertained with three different outcomes, i.e. perceived general health, need for recovery, and musculoskeletal complaints. Perceived general health (i.e. non musculoskeletal co-morbidity) was ascertained by 10 dichotomized questions about subjective health complaints, such as respiratory complaints, stomach complaints, regular headache, and tiredness. A sum score was calculated to represent the worker's actual health situation. This scale had a good internal scale reliability (Cronbach's α = 0.86) and test-retest reliability (Pearson's r = 0.76) [29]. Need for recovery was measured with 11 dichotomized questions assessing short-term health effects that reflect the worker's need for recovery at the end of a regular workday. These questions addressed items such as tiredness after work, fatigue, lack of concentration, putting interest in other people, the ability to recover from work, and the influence on work performance [30]. For both health endpoints subjects with a score above the median value were considered to have a high need for recovery and a moderate/bad general health. Musculoskeletal co-morbidity was defined as the presence of more than one complaint of the low back, shoulder/neck, or hand/wrist in the past 12 months. In the analysis three measures of MSD-comorbidity for each location of complaints were included, i.e. MSD-comorbidity for LBP consists of shoulder/neck pain (SNP) and/or hand/wrist pain (HWP) etc.

Health care use assessed by questions on the type of care-seeking by respondents for their musculoskeletal problems in the past 12 months. Medical care providers included a general practitioner, a specialist, a physiotherapist, or an occupational physician. All medical specialists, including orthopedic surgeons, other surgeons, and neurologists were grouped under specialty medical care. The category physiotherapists also included physical therapists and chiropractors.

For all employees retrospective data on absenteeism (occurrence, duration and diagnosis) in the past 12 months were available from the company sickness absence register. This register, kept in occupational health department, is mainly based on medical certifications issued by Social Insurance Institute, the official insurance covering body of most employees in the shipyard industry.

Three primary outcome measures for each type of MSD were defined: (i) a musculoskeletal complaint (low back, shoulder/neck, and hand/wrist) was defined as pain in the past 12 months, which had continued for at least a few hours during the past 12 months, (ii) a musculoskeletal complaint which led to an episode of sickness absence in the past 12 months, and (iii) a musculoskeletal complaint which led to health care use in the past 12 months.

### Statistical analysis

In the statistical analysis differences between normally distributed continuous variables were tested with the Student t-test and differences between categorical variables with the chi-square test (x^2^). Logistic regression analysis was performed to evaluate the influence of determinants on the occurrence and consequences of musculoskeletal complaints. Odds ratios (OR) with 95% confidence intervals were calculated as measure of association, adjusted for age and gender. For the initial selection of potential determiantns for musculoskeletal complaints univariate logistic regression analysis was used with of significance level of p < 0.10. Subsequently, all independent variables that showed significant associations were considered for inclusion into the multivariate logistic regression model and retained when significant at p < 0.05. These analyses were carried out separately for all three definitions of outcomes. The analyses on factors associated with seeking care and sickness absence were restricted to the subset of workers with musculoskeletal complaints. In the results, the final multivariate model is presented as well as the OR for other variables when included separately in this multivariate model. An OR above one indicates that the likelihood of symptoms, sick leave, or health care use is higher with the presence of the specified determinant. Data analyses were conducted by means of the SPSS for Windows 10.1.0 statistical package.

## Results

Table 1 shows the basic characteristics of the study population. The subjects consisted predominantly of blue collars with two or three year secondary school of technical expertise (60.3%). Only among white collar employees females were present (n = 56). In total, 25.2% of study population had never smoked, while another 15.5% were ex smokers. Smoking was associated with educational level and it was significantly more prevalent among blue collar jobs such as welders, sandblasters, and painters.

As expected, the self-reported physical workload and perceived exertion differed markedly between white and blue-collar employees. Although job control did not differ significantly between white and blue collar workers, it is worth mentioning that lower skill discretion was reported by white-collar workers and lower decision authority by blue-collar workers. White-collar workers reported higher job demands and a worse perceived general health compared with blue collars (Table 1).

The self-reported physical and psychosocial factors at work were partly determined by personal characteristics. Inverted trends of physical determinants, perceived exertion, and job control with age were present. A higher exposure to physical factors, perceived exertion, lower decision authority, and higher job demands were all associated with a higher need for recovery. A bad/moderate perceived general health was strongly associated with female gender, higher need for recovery and higher job demands.

Table 2 present the 12-month prevalences of musculoskeletal complaints and the occurrence of sickness absence and health care use. Low-back pain (LBP) was the most prevalent musculoskeletal complaint, reported by 36.8% of the subjects. Among workers with low back pain chronic pain (at least one month presence of complaint) was reported by 16.1% among blue-collar workers and 25.6% among white-collar workers (p = 0.052). White-collar workers also reported more complaints of shoulder/neck (SNP). Within the blue-collar workers hand/wrist complaints (HWP) were more prevalent in metal workers, while shoulder/neck and low back complaints were reported more often in welders, even though these differences did not reach statistical level of significance. Musculoskeletal co-morbidity was high. In the total population, one out of five reported at least two musculoskeletal complaints. Subjects with back pain more often reported shoulder/neck pain (34.4%) and hand/wrist pain (23.9%) than those without back pain (14.1% and 9.5%, respectively). Co-morbidity and chronicity of complaints were highly related. From those who reported at least two musculoskeletal complaints, chronicity of one or more complaints was reported by 28.7% and 19.1%, respectively.

Low-back pain resulted in higher absenteeism and health utilization than other musculoskeletal complaints (Table 2). Among workers with LBP, absenteeism was reported by 37.8% and 43.3% of white and blue collar workers, respectively. Among workers with SNP higher absenteeism was reported again by blue-collar workers (26.7%), mainly welders (41.2%). The same observation was made for absenteeism due to HWP with the highest proportion among metal workers (26.7%). Data from the accounting department showed that the total employment time reached 355000 working days in 2004 (1450 employees), while 2.75% was lost due to sickness absence. About 56% of the employees took at least period of one sick leave and MSDs accounted for 22.3% of total sick leaves and for 24% of total work days lost.

About 51% of workers with LBP went to physicians or other care givers. One out of three had visited more than one care giver and approximately four out of five of care seekers due to low back pain took a sick leave during last year. Care seeking and sick leave were less associated for shoulder/neck and hand/wrist complaints, especially among white collar workers.

In tables 3 to 5 the multivariate analyses for occurrence and consequences of low back, shoulder/neck, and hand/wrist complaints in the past 12 months are summarized. Ageing was associated with a higher occurrence of MSD complaints but older workers were less likely to take sick leave for low back and hand/wrist complaints. Females reported more complaints of shoulder/neck and hand/wrist, but among those women with these complaints care seeking and absenteeism was less compared with of men with the same complaints. Among those with complaints, blue collar workers and lower educated employees reported higher care seeking and absenteeism due to any complaint.

A high exposure to physical factors was associated mainly with the occurrence of complaints, and less with sickness absence and health care use. Psychosocial factors showed inconsistent associations with the outcomes under study. Low job control was related to more care-seeking due to hand/wrist complaints whereas high job demands was associated with fewer absences due to shoulder/neck pain.

A poor/moderate perceived health (i.e. non musculoskeletal co-morbidity) was strongly associated with the occurrence of MSD complaints, while a high need for recovery was associated only with LBP. Musculoskeletal co-morbidity was associated with more care seeking and higher absenteeism due to low back pain. Chronicity of complaints was the most important determinant of both health care utilization and absenteeism for any MSD complaint.

## Discussion

In this cross-sectional survey the burden of musculoskeletal disorders in Greek industry is reported for the first time. Various individual and work-related factors were associated with the occurrence of MSDs. Health care use and absenteeism were strongest influenced by chronicity of musculoskeletal complaints and comorbidity with other musculoskeletal complaints and, to a lesser extent, by work-related factors.

Some limitations of the study need to be considered in the interpretation of the result. First, this cross-sectional study does not permit conclusions as to the causality of the associations. Second, this study may suffer from information bias since most data were based on self-reports. The presence of recall bias may account for the associations between chronicity and care use when subject with more short, benign episodes of MSDs underestimate their actual care utilisation. Although this effect of recall bias cannot be excluded, our observations are in line with prospective studies on determinants of health care use [10,14]. This recall bias is less likely to play a role in the associations between work-related factors and MSDs and their consequences, since Toomingas and colleagues did not observe bias in self-reported physical exposure and pain [31]. Third, the interrelation between physical factors and psychosocial factors at work was high. As a consequence, in the multivariate analysis it is to some extent arbitrary which specific work-related determinant was included in the final model. Hence, the presented models cannot be used to target specific aspects of physical load or psychosocial load. In addition, the inclusion of correlated variables in a multivariate analysis may result in lower ORs.

The descriptive part of the study demonstrated high prevalences of complaints of back, neck/shoulder, and hand/wrist. Prevalences of MSDs between 20–60% have been reported for carpenters, painters, and metal workers [12,18,32-34]. White-collar workers had a higher prevalence of MSD complaints than the blue-collar workers, which has been observed before for complaints of shoulder and neck [35-37]. This finding may be partly explained by the shift towards white-collar jobs of medically unfit blue-collar employees, which has been regular practice in this company during the past decade.

A substantial proportion of workers with MSD, approximately 39–53%, sought medical care for their complaints in the past 12 months. The mechanisms underlying decisions to seek medical care are not well understood. A study in scaffolders identified chronic and severe pain as primary factors that determined specific type of care-seeking due to back pain among industry workers [11]. It has been pointed out in previous studies that differences in health care systems and cross-cultural factors will influence the type of medical care sought [38,39]. In other studies care-seeking was determined by physical and psychosocial occupational factors, complaint-related characteristics and musculoskeletal comorbidity [14,16,40]. In our study population, chronicity and musculoskeletal comorbidity has the strongest influence on health care use, confirming results from other studies [11,14,16,40]. An interesting finding was that white-collar workers reported significantly more MSD complaints, but sought care less often than blue-collar workers. This may be due to the fact that a white collar employee may cope better with duties at work in contrast to the physical demands of blue collar employees [36]. This mechanism may also explain why females reported less care seeking, especially for low back and shoulder neck complaints, since the job content of the women workers in this setting was less demanding for low back and shoulder/neck compared with the hand/wrist (i.e. PC work) [41]. Living with others and having kids was also related to higher care utilization. Perhaps the need for care and rehabilitation is higher when home demands are increased.

A substantial proportion of the workers also took a period of sickleave, often overlapping with medical care seeking. In this study population in a shipyard about 0.7% of the total working time was lost in a year due to musculoskeletal disorders, equalling approximately half a million Euros. The decision to take sick leave is probably more complex than care seeking. Our results confirmed that sickness absence is much more frequent for back pain and the occurrence of sickleave was comparable with study populations in similar settings [11,42]. The strongest associations of absenteeism, similar to health care use, were shown with chronicity and comorbidity of complaints. Complaints-related aspects have been reported to be more strongly associated with sick leave than work-related aspects [40]. In our study, musculoskeletal comorbidity was strongest associated with absenteeism due to low back pain. Since sick leaves are far more frequent for low back pain than other MSDs, a sickleave due to LBP might also be beneficial for the recovery of other musculoskeletal complaints.

Older employees reported more complaints but they took less sick leaves, even though in our study this was not a consistent finding across different musculoskeletal complaints. It is reported that the frequency of sickness absence among older workers is lower, but that the average duration of a sickleave spell may be longer [32,42,43]. In general, blue-collar workers and lower educated employees reported higher absenteeism but in the analysis this could not be attributed to more physically demanding job activities. As stated before, the aspects of physical load were measured rather crudely on a four-point scale and, thus, these variables will lack discriminatory power. In addition, the patterns of physical load were distinctively different for blue-collar and white-collar jobs and for men and women. In the statistical analysis it could not be ascertained whether the observed impact of job type and gender on sickness absence (and health care use) was partly due to differences in physical load.

In general, we found weak associations between psychosocial factors at work and subjective health complaints with absenteeism (mainly for shoulder/neck), while other studies have shown various effects [9,20,44-46]. However, one has to bear in mind that in the current study only a limited number of psychosocial aspects at work were taken into consideration. Given the importance of chronicity of complaints for care seeking and sickness absence, more attention is needed to those factors that determine the transition from acute to chronic MSDs, especially individual psychological traits. A disadvantage of this occupational study is that psychological factors were not addressed and, thus, their potential influence on absenteeism and care seeking could not be established.

## Conclusion

In conclusion, several individual and work-related physical and psychosocial factors were associated with the occurrence of MSDs. Health care use and absenteeism were strongest influenced by chronicity of musculoskeletal complaints and comorbidity with other musculoskeletal complaints and, to a lesser extent, by work-related factors. Among those with musculoskeletal complaints, more demanding job tasks seem to be related with the decision to take sick leave and seek care as was indicated by more care seeking and absenteeism among blue collar workers. In programmes aimed at preventing the unfavourable consequences of MSD in terms of sickness absence and health care use it is important to identify the (individual) factors that determine the development of chronicity of complaints. These factors may differ from the well-know risk factors for the occurrence of MSD that are targeted in primary prevention.

## Competing interests

The author(s) declare that they have no competing interests.

## Authors' contributions

ECA designed the study protocol, managed the co-ordination, performed the statistical analysis, drafted and revised the manuscript.

DT managed the data collection and participated in the analysis.

EK participated in the analysis and in drafting the manuscript.

AB participated in drafting and revised critically the manuscript.

All authors read and approved the final manuscript.

## Pre-publication history

The pre-publication history for this paper can be accessed here:



## Figures and Tables

**Table 1:** Individual characteristics and self-reported determinants for musculoskeletal disorders among shipyard employees (n = 853)

	White collar workers (n = 229)	Blue collar worker (n = 624)
Age (y, mean (SD))	36.8 (9.1)	38.7 (9.5)
Height (cm, mean(SD))	174.9 (8.8)	176.1 (6.5)
Body mass index		
(kg/m^2^, mean (SD))	26.0 (4.8)	27.4 (4.0)
Educational level (%) *		
Higher	46.9	3.4
Technical	29.6	60.3
Basic	23.5	36.3
Duration of employment *		
(y, mean (SD))	9.5 (8.8)	16.0 (10.0)
Family situation (%) *		
Alone	24.6	12.4
Relatives/friends	75.4	87.6
Manual material handling (%) *	6.0	21.8
Strenuous shoulder/hand movements (%) *	34.6	66.7
Strenuous (awkward) postures (%) *	17.3	53.6
Perceived exertion (score, mean (SD)) *	11.05 (3.65)	14.01 (2.72)
Psychosocial load (score, mean (SD)):		
Job control	41.24 (24.04)	40.53 (22.25)
Work demands *	44.36 (22.40)	36.47 (21.36)
General health		
Need for recovery (score, mean (SD)	40.55 (28.52)	39.41 (26.85)
Perceived general health (score, mean (SD) *	21.72 (21.43)	17.38 (18.97)
Musculoskeletal co-morbidity (%) *	24.1	16.3

* x^2 ^or t-test, p < 0.05

**Table 2:** Prevalence of symptoms and consequences of musculoskeletal disorders in the past 12 months among shipyard employees (n = 853)

	White collars (n = 229)	Metal workers (n = 287)	Welders (n = 93)	Other blue collars (n = 244)
Low back pain (%)				
12 months prevalence	39.3	33	39.8	37.9
sickleave in past 12 months	14.8	12.2	17.2	18.9
health care in past 12 months	17.9	17	20.4	21.4
Shoulder/neck pain (%)				
12 months prevalence *	27.9	14.6	18.3	25.2
sickleave in past 12 months	4.4	4.4	7.5	5.0
health care in past 12 months	11.8	7.5	8.6	8.0
Hand/wrist pain (%)				
12 months prevalence	17.0	15.3	10.8	13.4
sickleave in past 12 months	2.6	4.1	2.2	2.9
health care in past 12 months	6.6	7.1	5.4	8.4

* x^2 ^test, p < 0.05

**Table 3:** Determinants for the occurrence of low back pain, sick leave and health care use in the past 12 months among shipyard employees

Self reported risk factors	Low back pain (n = 853)		Health care use (n = 314)		Sick leave (n = 314)	
	OR^§^	95% CI	OR^§^	95% CI	OR^§^	95% CI
Age						
≤ 30	1.00		1.00		1.00	
31–44	1.53*	1.04 2.25	0.85	0.46 1.58	0.89	0.46 1.71
45 ≥	1.48	0.97 2.26	1.06	0.54 2.08	0.50	0.24 1.03
Females	1.19	0.62 2.28	0.64	0.24 1.71	0.34	0.11 1.02
Body mass index >30 kg/m2	0.81	0.55 1.20	1.06	0.59 1.91	1.32	0.71 2.45
Living alone	1.04	0.68 1.59	0.87	0.46 1.67	1.47	0.75 2.87
Kids	1.12	0.80 1.57	0.93	0.56 1.54	0.70	0.41 1.19
Blue collars	0.84	057 1.23	1.31	0.74 2.32	1.28	0.72 2.27
Low level of education	1.09	0.70 1.68	0.97	0.49 1.91	1.34	0.64 2.81
Supervising	1.00	0.73 1.39	1.09	0.68 1.75	0.56*	0.32 0.99
Manual material handling	1.55*	1.02 2.36	1.13	0.63 2.02	1.20	0.65 2.20
Strenuous shoulder movements	1.21	0.87 1.67	1.23	0.76 2.00	1.25	0.74 2.09
Strenuous awkward postures	1.35	0.96 1.88	1.41	0.85 2.32	1.42	0.84 2.40
High perceived exertion	1.34	0.98 1.84	1.45	0.90 2.32	1.57	0.96 2.58
Low job control	1.07	0.79 1.46	1.18	0.74 1.88	1.37	0.84 2.24
High job demands	1.00	0.72 1.38	0.89	0.55 1.44	1.11	0.66 1.87
High need for recovery	2.11*	1.49 2.98	1.25	0.76 2.08	0.95	0.56 1.62
Bad/moderate perceived general health	1.76*	1.25 2.48	1.03	0.64 1.66	0.92	0.55 1.55
MSD comorbidity		-				
LBP			1.00		1.00	
LBP and HWP			1.29	0.61 2.75	1.53	0.66 3.59
LBP and SNP			1.33	0.73 2.41	1.74	0.89 3.39
ALL			1.10	0.53 2.29	2.78*	1.17 6.60
Chronic complaint		-	2.44*	1.23 4.83	2.36*	1.19 4.70

^§^Significant factors constituting the multivariate model are indicated by '*' (i.e., P < 0.05). For other factors, the OR (prevalence ratio) is presented when including this factor in the multivariate model.

CI = confidence interval.

**Table 4:** Determinants for the occurrence of hand wrist pain, sick leave and health care use in the past 12 months among shipyard employees

Self reported risk factors	Hand wrist pain (n = 853)		Health care use (n = 126)		Sick leave (n = 126)	
	OR^§^	95% CI	OR^§^	95% CI	OR^§^	95% CI
Age						
≤ 30	1.00		1.00		1.00	
31–44	1.25	0.74 2.12	3.51*	1.19 10.34	0.29*	0.10 0.83
45 ≥	1.64	0.92 2.91	0.64	0.24 1.73	0.36	0.12 1.07
Females	3.82*	1.93 7.58	0.29	0.06 1.45	0.81	0.23 2.85
Body mass index >30 kg/m2	1.39	0.85 2.26	1.59	0.62 4.10	1.00	0.33 3.05
Living alone	0.75	0.41 1.37	0.25*	0.07 0.92	0.90	0.24 3.38
Kids	1.15	0.73 1.82	1.86	0.72 4.82	1.21	0.42 3.46
Blue collars	1.42	0.79 2.55	9.45*	2.20 40.51	1.87	0.32 0.84
Low level of education	1.06	0.59 1.91	3.72	0.77 18.02	3.10	0.59 6.24
Supervising	1.34	0.87 2.07	1.00	0.43 2.34	0.76	0.31 1.91
Manual material handling	1.99*	1.18 3.35	0.60	0.22 1.66	0.47	0.14 1.54
Strenuous shoulder movements	1.39	0.89 2.18	0.17*	0.06 0.50	1.36	0.44 4.17
Strenuous awkward postures	1.32	0.83 2.10	1.07	0.39 2.96	1.01	0.36 2.85
High perceived exertion	1.18	0.76 1.82	0.98	0.40 2.45	1.34	0.50 3.58
Low job control	1.06	0.70 1.61	1.51	0.65 3.47	0.82	0.32 2.07
High job demands	0.76	0.50 1.18	0.89	0.38 2.06	1.45	0.54 3.88
High need for recovery	1.01	0.63 1.61	0.60	0.25 1.44	0.57	0.22 1.47
Bad/moderate perceived general health	2.52*	1.64 3.87	0.85	0.36 2.00	0.86	0.33 2.27
MSD comorbidity						
HWP			1.00		1.00	
HWP and LBP			1.17	0.38 3.56	0.41	0.12 1.42
HWP and SNP			5.27*	1.09 25.39	0.74	0.18 2.99
ALL			1.36	0.46 4.08	0.61	1.19 1.90
Chronic complaint			2.53	0.98 6.51	1.62	0.60 4.37

^§^Significant factors constituting the multivariate model are indicated by '*' (i.e., P < 0.05). For other factors, the OR (prevalence ratio) is presented when including this factor in the multivariate model.

CI = confidence interval.

**Table 5:** determinants for the occurrence of shoulder neck pain, sick leave and health care use in the past 12 months among shipyard employees

Self reported risk factors	shoulder neck pain (n = 853)		Health care use (n = 184)		Sick leave (n = 184)	
	OR^§^	95% CI	OR^§^	95% CI	OR^§^	95% CI
Age						
≤ 30	1.00		1.00		1.00	
31–44	1.61*	1.04 2.50	1.40	0.62 3.15	0.75	0.25 2.30
45 ≥	1.94*	1.21 3.11	1.36	0.58 3.19	1.49	0.43 5.13
Females	1.70	0.90 3.11	0.55	0.19 1.58	0.66	0.16 2.72
Body mass index >30 kg/m2	0.84	0.54 1.31	1.27	0.58 2.75	0.56	0.18 1.76
Living alone	1.14	0.72 1.82	0.79	0.34 1.83	0.50	0.15 1.70
Kids	0.83	0.57 1.22	1.31	0.66 2.61	0.27*	0.10 0.75
Blue collars	0.72	0.47 1.09	0.78	0.37 1.62	2.10	0.66 6.61
Low level of education	0.99	0.61 1.60	1.12	0.48 2.60	1.68	0.42 6.62
Supervising	0.93	0.64 1.34	0.74	0.39 1.42	0.69	0.27 1.77
Manual material handling	0.75	0.45 1.25	1.07	0.42 2.70	1.04	0.33 3.26
Strenuous shoulder movements	1.04	0.72 1.49	1.08	0.57 2.07	3.87*	1.50 9.99
Strenuous awkward postures	1.15	0.79 1.67	0.80	0.41 1.58	1.15	0.43 3.09
High perceived exertion	0.87	0.60 1.24	1.18	0.63 2.21	1.60	0.68 3.79
Low job control	1.10	0.78 1.56	1.12	0.60 2.08	1.32	0.59 2.96
High job demands	0.93	0.65 1.34	0.61	0.32 1.16	0.41*	0.17 0.96
High need for recovery	1.28	0.86 1.89	0.95	0.49 1.85	1.79	0.71 4.55
Bad/moderate perceived general health	3.63*	2.55 5.16	1.34	0.70 2.58	2.28	0.91 5.69
MSD comorbidity		-				
SNP			1.00		1.00	
SNP and LBP			1.17	0.56 2.48	0.91	0.36 2.34
SNP and HWP			3.31	0.96 11.40	1.08	0.26 4.49
ALL			1.42	0.59 3.41	0.37	0.09 1.44
Chronic complaint		-	2.85*	1.52 5.35	3.42*	1.38 8.46

^§^Significant factors constituting the multivariate model are indicated by '*' (i.e., P < 0.05). For other factors, the OR (prevalence ratio) is presented when including this factor in the multivariate model.

CI = confidence interval.
